# Every gene everywhere all at once: High-precision measurement of 3D chromosome architecture with single-cell Hi-C

**DOI:** 10.3389/fmolb.2022.959688

**Published:** 2022-10-06

**Authors:** Yi Chi, Jenny Shi, Dong Xing, Longzhi Tan

**Affiliations:** ^1^ Biomedical Pioneering Innovation Center, Peking University, Beijing, China; ^2^ Innovation Center for Genomics, Peking University, Beijing, China; ^3^ Department of Neurobiology, Stanford University, Stanford, CA, United States; ^4^ Department of Chemistry, Stanford University, Stanford, CA, United States; ^5^ Department of Bioengineering, Stanford University, Stanford, CA, United States

**Keywords:** genome architecture, epigenetic regulation, spatial omics, DNA folding, chromatin structure

## Abstract

The three-dimensional (3D) structure of chromosomes influences essential biological processes such as gene expression, genome replication, and DNA damage repair and has been implicated in many developmental and degenerative diseases. In the past two centuries, two complementary genres of technology—microscopy, such as fluorescence *in situ* hybridization (FISH), and biochemistry, such as chromosome conformation capture (3C or Hi-C)—have revealed general principles of chromosome folding in the cell nucleus. However, the extraordinary complexity and cell-to-cell variability of the chromosome structure necessitate new tools with genome-wide coverage and single-cell precision. In the past decade, single-cell Hi-C emerges as a new approach that builds upon yet conceptually differs from bulk Hi-C assays. Instead of measuring population-averaged statistical properties of chromosome folding, single-cell Hi-C works as a proximity-based “biochemical microscope” that measures actual 3D structures of individual genomes, revealing features hidden in bulk Hi-C such as radial organization, multi-way interactions, and chromosome intermingling. Single-cell Hi-C has been used to study highly dynamic processes such as the cell cycle, cell-type-specific chromosome architecture (“structure types”), and structure–expression interplay, deepening our understanding of DNA organization and function.

## 1 Introduction

How DNA folds in the nucleus is a fundamental question in biology. The spatial separation of the euchromatin and heterochromatin has been observed since the early age of microscopy. In the 1980s, the development of DNA fluorescence *in situ* hybridization (FISH) validated the hypothesis of chromosome territories and revived the study of nuclear architecture (Jerkovic’ and Cavalli, 2021). FISH and fluorescent protein imaging brought about fruitful research on the behavior of genomic loci; however, their genomic coverage and resolution remained a bottleneck for further exploration along the path (Ulianov et al., 2017).

**FIGURE 1:**
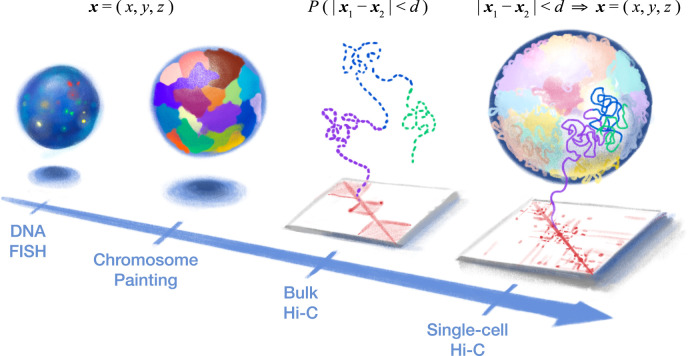
Single-cell Hi-C provides a holistic high-resolution view into the 3D structure of our genetic blueprint. (Left) DNA fluorescence *in situ* hybridization (FISH) provided the first look into genome organization in the cell nucleus by directly measuring 3D coordinates (i.e., *x*, *y*, and *z*) of various genomic loci (or entire chromosomes, in the case of chromosome painting) but is limited by the optical resolution and spectrum (i.e., the number of loci). (Right) Chromosome conformation capture (3C or Hi-C) indirectly measures nuclear architecture through 3D proximity between genomic loci (i.e., “contact map”). Bulk Hi-C measures the average 3D proximity (probability (*P*) that two loci are within a certain 3D distance (*d*)) among a large population of cells and, therefore, cannot produce true 3D structures (dashed line). In particular, the “all-to-all” inter-chromosomal contacts in bulk Hi-C provide conflicting spatial constraints, while chromatin domains (three are depicted here) would be seemingly isolated from each other. In contrast, single-cell Hi-C offers a new concept of a “biochemical microscope.” 3D proximity of a single cell can be converted into actual 3D coordinates of the whole genome, yielding high-resolution structures without the need for specialized equipment.

The development of genome-wide chromatin architecture technologies has brought the field of 3D genomics into a new era (Figure 1). At the single-cell level, these technologies primarily fall into two categories: sequencing-based methods and imaging-based methods (Ulianov and Razin, 2021). Imaging-based methods directly measure the 3D coordinates of labeled genomic loci. These methods include chromatin tracing (Wang et al., 2016; Bintu et al., 2018; Nir et al., 2018), ORCA (Mateo et al., 2019), Hi-M (Cardozo Gizzi et al., 2019), DNA-MERFISH (Su et al., 2020), DNA seqFISH+ (Takei et al., 2021), OligoFISSEQ (Nguyen et al., 2020), and IGS (Payne et al., 2021).

In contrast, sequencing-based methods measure 3D spatial proximity between genomic loci, producing “chromatin contact maps” that indirectly reflect the relationship between 3D coordinates. The majority of these methods are based on the digestion of DNA followed by proximity ligation—originally pioneered by bulk chromosome conformation capture assays (3C (Dekker et al., 2002) or Hi-C (Lieberman-Aiden et al., 2009))—yielding artificial ligation junctions (“chromatin contacts”) between genomic loci that are far away along the linear sequence but nearby in 3D. Therefore, here, we refer to them as “single-cell Hi-C” (scHi-C) methods (Nagano et al., 2013, 2017; Flyamer et al., 2017; Ramani et al., 2017; Stevens et al., 2017; Tan et al., 2018; Lee et al., 2019; Li et al., 2019). Other sequencing-based methods are ligation-free—such as scSPRITE (Arrastia et al., 2021), GAM (Beagrie et al., 2017), and immunoGAM (Winick-Ng et al., 2021).

These technologies greatly improved our understanding of both higher-order and fine-scale chromosome structures (Fraser et al., 2015). Hi-C strongly supported the existence of chromosome territories because contacts are highly enriched within each chromosome (“intra-chromosome”). Spatial segregation of the euchromatin and heterochromatin manifests as a “plaid” (or “checkerboard”) pattern in the contact map—a phenomenon termed “chromatin A/B compartmentalization” (Lieberman-Aiden et al., 2009). Further studies have shown that sub-compartments exist within the primary A (euchromatin) and B (heterochromatin) compartments, corresponding to spatial segregation of additional epigenetic marks (Rao et al., 2014). The driving force behind compartmentalization might be the phase separation of the chromatin (Falk et al., 2019).

On finer scales, chromatin domains [also known as “topological-associated domains” (TADs)] were discovered as sub-megabase (Mb) chromatin structures (Dixon et al., 2012; Nora et al., 2012; Sexton et al., 2012). At this scale, chromatin loops (also known as “dots” in the contact map), “stripes,” and more complex patterns also emerged from high-resolution Hi-C (Rao et al., 2014) and Micro-C data (Hsieh et al., 2020; Krietenstein et al., 2020; Akgol Oksuz et al., 2021). Among them, loops manifest as pixels in the contact map with higher contact frequencies than neighboring pixels and are usually found at domain boundaries (i.e., at the corner of diagonal “squares” in the contact map). Some believed loops to be stable, but recent studies have preferred a more transient view (Mirny and Dekker, 2021).

In this mini review, we focus on proximity ligation-based DNA sequencing methods for measuring single-cell nuclear architecture. We first introduce current technologies and then review discoveries made by applying them to different biological systems—especially those achievable only with single-cell methods.

## 2 Current single-cell Hi-C methods

In 2013, Nagano et al. (2013) pioneered an incredible feat of bringing Hi-C—which normally required a large number of cells—to the single-cell level. Although key features of bulk Hi-C—such as the enrichment of intra-chromosomal contacts (i.e., chromosome territory)—are preserved, single-cell Hi-C (scHi-C) contact maps uncovered extraordinary cell-to-cell variability of the genome structure. In particular, the highly variable, “patchy” inter-chromosomal contacts are in sharp contrast to the much smoother “all-to-all” contacts in bulk Hi-C. Although the diploid nature of our genome prevented the 3D reconstruction of autosomes (because each contact map was a mixture of different chromosomal copies) at the time, Nagano *et al.* solved the structure of the (single-copy) male X chromosome. The spatial resolution of these structures was limited by the biochemistry of the time.

In 2017, multiple groups optimized scHi-C technologies. Flyamer et al. (2017) improved the sensitivity of scHi-C and applied it to the oocyte-to-zygote transition in mice. Nagano et al. (2017) redesigned their assay to yield more contacts and better throughput and applied it to the cell cycle in mouse embryonic stem cells (mESCs). Stevens et al. (2017) solved the first 3D genome structure of a single mammalian cell, taking advantage of a special haploid mESC line, thus circumventing the challenge of the normally diploid genome. In sci-Hi-C, Ramani et al. (2017) used combinatorial indexing to achieve high throughput. Single-cell Hi-C methods until 2017 have been reviewed in detail by Ulianov et al. (2017).

In 2018, Tan et al. (2018) solved the first 3D genome structure of the human genome [and the diploid mouse genome using a new method termed Dip-C—which combined improved biochemical sensitivity including a transposon-based whole-genome amplification (WGA) method META] and an algorithm to solve the challenge of the diploid genome by imputing haplotypes from sparse data—and applied Dip-C to the human blood.

scHi-C methods continued to grow in the past years. In 2019, methyl-Hi-C (Li et al., 2019) and sn-m3c-Seq (Lee et al., 2019) combined proximity ligation and whole-genome bisulfite sequencing (WGBS) to simultaneously profile DNA methylation and chromatin architecture.

Sensitivity and scalability are two major criteria to compare the performance of scHi-C methods (Galitsyna and Gelfand, 2021; Zhou et al., 2021). Scalability has been improved by automation (Nagano et al., 2017; Tan et al., 2021) and split-pool barcoding (Ramani et al., 2017). Sensitivity has been improved by better WGA methods (Flyamer et al., 2017; Tan et al., 2018) and one-step library preparation with transposase (Nagano et al., 2017; Stevens et al., 2017; Tan et al., 2018). However, a systematic, uniform comparison of scHi-C methods that takes into account the cell type, ploidy, sequencing depth, and data processing is still lacking (Lando et al., 2018).

## 3 Revising Hi-C concepts in single cells

### 3.1 Single-cell chromatin domains

Originally defined as contact-rich “squares” along the diagonal line in bulk Hi-C data, chromatin domains [also known as topologically associating domains (TADs)] are characterized by increased contacts within each domain (“intra-domain”) and decreased contacts between domains (“inter-domain”). This appealing feature seems to suggest a role in genome function—for example, to confer specificity of enhancer–promoter interactions by partitioning regulatory elements into different domains (van Arensbergen et al., 2014); however, evidence both for and against this proposed function has accumulated (Nora et al., 2017; Rao et al., 2017; Ghavi-Helm et al., 2019; Zuin et al., 2022). Therefore, the origin and function of chromatin domains are still under debate.

Chromatin domains are usually illustrated based on bulk Hi-C as “globules” of DNA with the two boundary loci stably contacting (Figure 1 middle); however, an alternative explanation is that domains are just population-averaged contact preferences with no discernable structures in single cells. The true extent of cell-to-cell variability is masked in bulk Hi-C data.

Single-cell Hi-C (scHi-C) shed light on those questions. As expected, the ensemble average of scHi-C contact maps reproduced all bulk Hi-C features—including the intensity and position of each chromatin domain “square” (Nagano et al., 2013). In addition, within each cell, the average contact profile (“pile-up”) of bulk Hi-C domains and bulk Hi-C loops showed expected enrichment of contacts within domains and at loop anchors (Flyamer et al., 2017; Gassler et al., 2017).

Cluster of contacts were prominently detected in scHi-C contact maps; however, boundaries of these “single-cell chromatin domains” [sometimes referred to as “TAD-like structures” (TDLs)] were highly variable across cells and did not always coincide with bulk Hi-C domains (Flyamer et al., 2017). Since then, additional algorithms have been developed to systematically annotate single-cell domains, and these domains were found to prefer common boundaries and correlate with epigenetic marks and transcription (Zhou et al., 2019; Li et al., 2021; Zhang et al., 2021). Cell-to-cell variability of chromatin domains has been confirmed with imaging-based methods, which provided additional mechanistic insights (Bintu et al., 2018; Finn et al., 2019; Luppino et al., 2020; Szabo et al., 2020)—for example, cohesin seemed dispensable for single-cell domains (Bintu et al., 2018) but is necessary for bulk Hi-C domains (Rao et al., 2017; Schwarzer et al., 2017).

What is the nature of single-cell chromatin domains? Do they have the same origin as bulk Hi-C domains? Modeling suggested that domain structures called “proto-TADs” naturally emerged from random chromatin polymers confined to a volume (Gürsoy et al., 2014) and that entropy might play a role (Vasquez et al., 2016). The theory of loop extrusion was also used to explain the heterogeneity of single-cell domains, where many chromatin loops are dynamically created and extruded by loop-extruding factors (LEFs) translocating along the chromatin fiber (Flyamer et al., 2017; Liang and Perez-Rathke, 2021; Yu et al., 2021; Dequeker et al., 2022; Li et al., 2022). Questions about single-cell domains have not been settled yet. Care must be taken when studying this question because chromatin domains have developed into a complex concept with different meanings and origins (Beagan and Phillips-Cremins, 2020).

### 3.2 Single-cell chromatin compartments

Unlike the “globular” domains, chromatin compartmentalization does not imply a specific physical structure. Chromatin compartments were originally defined from a statistical perspective: genomic loci were divided based on their chromosome-wide contact profiles into two mutually anti-correlated types—“compartment A” and “compartment B.”

Single-cell Hi-C (scHi-C) allowed bulk Hi-C A/B compartments to be visualized in 3D. When scHi-C 3D structures were colored by bulk-defined compartments, the A and B compartments were relatively segregated (Stevens et al., 2017; Tan et al., 2018). This segregation was consistent with imaging-based studies, where single chromosomes showed polarized configuration of compartments (Wang et al., 2016); later, results with higher resolution found more variable spatial arrangements like “sandwiches” and more overlaps between A and B (Su et al., 2020).

Coloring 3D structures with bulk-defined compartments has limitations because single cells—especially cells of different types—can have different epigenomic profiles and, therefore, different chromatin compartments (Su et al., 2020; Ulianov and Razin, 2021). This is even more necessary, considering exceptions to the traditional A/B classification [e.g., the intermediate (“I-type”) compartment, splicing factories, and polycomb-repressed H3K27me3 regions] (Mirny and Dekker, 2021).

The *de novo* compartment calling directly from single cells is conceptually challenging because bulk Hi-C algorithms rely on cross-correlations between population-averaged long-range contact profiles. Long-range contacts are sparse in single cells because of the sensitivity of contact detection and, more fundamentally, because each genomic locus can only have a limited number of 3D neighbors in each cell.

To accomplish *de novo* compartment annotation in single cells, a practical solution is to define each genomic locus’s “single-cell compartment score” as the average value of some property of its 3D neighbors, instead of binary A/B categorization. For example, in defining the “A-association score,” the property of choice was the bulk Hi-C A/B compartment (Nagano et al., 2017), whereas in defining “scA/B” values, the property of choice was the frequency of CpG dinucleotides (which is derived from the genome sequence itself rather than from bulk Hi-C) (Tan et al., 2018).

These single-cell compartment scores are essentially the result of 3D “diffusion”/“smoothing” of certain genomic properties. Globally, these metrics were highly correlated with bulk Hi-C annotation (Nagano et al., 2017); however, their cell-to-cell differences were found to convey critical cell identity information, which can be extracted by the dimension-reduction algorithms to tell apart cell types without additional information (i.e., “3D genome structure typing”) (Tan et al., 2018). In addition, developmental switching of single-cell compartment scores correlates with changes in transcription (Tan et al., 2021).

More recent algorithms include imputation based on hypergraph representation learning, which enabled direct identification of single-cell compartments (Zhang et al., 2021). Non-backtracking walks were also reported to find compartments in sparse single-cell data (Polovnikov et al., 2020). It would be worth comparing these methods, especially on large datasets with complex cell types.

## 4 Dynamics and cell-type specificity of chromatin structures

By the time this mini review was written, no more than 20 single-cell Hi-C (scHi-C) studies have been published in peer-reviewed journals. A fair proportion of them studied cell lines or their synthetic mixture (Nagano et al., 2013, 2017; Ramani et al., 2017; Stevens et al., 2017; Li et al., 2019; Kim et al., 2020; Ulianov et al., 2021; Pang et al., 2022), while the application of scHi-C to primary tissues has been growing recently. Researchers have exploited different technical features of scHi-C according to the biological systems they studied.

First, scHi-C has the unique advantage of straightforward reconstruction of 3D models (Figure 1 right) and can, therefore, reveal structural features unattainable from bulk Hi-C contact maps. This makes scHi-C a new approach to uncover, visualize, and quantify a variety of nuclear structures. Interesting examples include the Rabl configuration in many species (Stevens et al., 2017; Tan et al., 2018; Zhou et al., 2019), “inside-out” configuration of retinal rods in nocturnal animals (Tan et al., 2019), inter-chromosomal gene and enhancer hubs (also known as “Greek Islands”) in olfactory sensory neurons (Tan et al., 2019), and the “compact silent center” (CSC) in rice (Zhou et al., 2019).

Second, dynamic changes can be reconstructed despite the snapshot nature of Hi-C when enough cells in different states of a process are covered. A pioneering example is *in silico* cell cycle phasing of mouse embryonic stem cells (mESCs) based on scHi-C contact maps (Nagano et al., 2017). Intensities of compartments, domains, and loops were found to change continually along the cell cycle. Another example is early embryonic development, where bulk Hi-C observed early relaxed chromatin, followed by a prolonged process of higher-order structure establishment in succession (Du et al., 2017; Ke et al., 2017). scHi-C identified domains and loops as early as G1-phase zygotes (Flyamer et al., 2017; Gassler et al., 2017) but agreed that these structures were much weaker than in later stages (Zheng and Xie, 2019). A more recent study with a larger sample size found that domains could be divided into several clusters according to their temporal patterns, such as early parental-specific domains and *de novo* domains arising at later stages (Collombet et al., 2020). Later in the developmental process, the trajectory of mESC differentiation was analyzed using “contact decay profiles” (CDPs) of sci-Hi-C contact maps (Bonora et al., 2021).

Third, similar to other single-cell technologies that have been used to create a human cell atlas, scHi-C can capture many cell types at once from complex tissues. This is particularly useful when direct cell sorting is impossible because of a lack of prior knowledge (Schwartzman and Tanay, 2015). Applying scHi-C to the human and mouse brain, researchers demonstrated this “atlasing” capability by showing that major cell types can be delineated from chromosome conformation alone (e.g., clustering based on scA/B)—albeit less sensitive than transcriptome- or methylome-based clustering (Lee et al., 2019; Tan et al., 2021). This implies that cell identity is at least partially encoded in the chromatin structure (Winick-Ng et al., 2021). The chromatin structure was also found to correlate with gene expression (Tan et al., 2021) and the epigenome at single-cell resolution (Lee et al., 2019), which is expected because “omic” data were usually correlated with each other (Boix et al., 2021). More interestingly, scHi-C uncovered a new mode of neuron maturation—the radial reconfiguration of certain genes (Tan et al., 2019, 2021). This discovery again demonstrates that new epigenome technologies provide a new understanding of the cell activity from a different angle—in the case of scHi-C, the new angle is from “space.”

## 5 Perspective

Single-cell Hi-C (scHi-C) is, in theory, a distance geometrical measurement of the chromatin, while microscopy is mostly Cartesian. scHi-C and microscopy tend to corroborate each other (Bintu et al., 2018) but also have important differences. The capture radius of the crosslinking-ligation chemistry in Hi-C is relatively short (presumably 10–100 nm) compared to the diffraction limit of conventional optical microscopy (Maslova and Krasikova, 2021). In addition, Hi-C does not measure chirality and, therefore, cannot differentiate enantiomorphs (i.e., mirror images) (Acemel et al., 2016; Xie et al., 2017). Combining scHi-C and microscopy will generate more accurate 3D structures using chirality and long-distance information. Microscopy followed by Hi-C has been used in some studies (Stevens et al., 2017; Lando et al., 2018).

Recent advances in bulk Hi-C chemistry—including new digestion enzymes (Hsieh et al., 2015, 2016) and crosslinking reagents (You et al., 2021)—are worth adopting for single cells. The use of micrococcal nuclease (MNase) will, in principle, increase the number of possible contacts and, therefore, alleviate the data sparsity problem (Galitsyna and Gelfand, 2021). In addition, scHi-C chemistry will also benefit from advances in whole-genome amplification (WGA). For example, the phi29 DNA polymerase in multiple displacement amplification (MDA) was reported to generate artifactual contacts in scHi-C (Ulianov et al., 2021); less biased WGA methods (Chen et al., 2017) and filtering algorithms may increase the accuracy of scHi-C.

One unanswered question in the field of 3D genomics is the function of genome folding: how do genome folding and gene expression influence each other? Functional perturbations—such as the depletion of a few key structural proteins—gave us a limited overview of this structure–function relationship (Rao et al., 2017). Genome editing studies (Zuin et al., 2022) and simultaneous multi-omic measurements may give us a more quantitative rulebook of genome organization. Development of new 3D multi-omic methods (Cardozo Gizzi et al., 2019; Nguyen et al., 2020; Payne et al., 2021; Takei et al., 2021) is, therefore, an exciting next step.
